# Subchronic Toxicity of Microcystin-LR on Young Frogs (*Xenopus laevis*) and Their Gut Microbiota

**DOI:** 10.3389/fmicb.2022.895383

**Published:** 2022-05-12

**Authors:** Jinjin Li, Hongzhao Sun, Chun Wang, Shangchun Li, Yunfei Cai

**Affiliations:** ^1^School of Life Sciences, Qilu Normal University, Jinan, China; ^2^School of Ecology and Environment, Beijing Technology and Business University, Beijing, China; ^3^School of Public Health, Southwest Medical University, Luzhou, China

**Keywords:** *Xenopus laevis*, cyanotoxin, gut microbiota, inflammation, ecological security assessment

## Abstract

Although toxic effects of microcystins (MCs) in mammals and fish have been extensively studied, the effects of MCs on the immune system and gut microbiota of amphibians have not received sufficient attention. As MCs cause general damage to the vertebrate liver and immune system and trigger an inflammatory response, and the gut microbiota is closely related to host metabolism and immunity, we speculated that MCs can cause changes in the immune system and gut microbiota of amphibians. To verify this, we examined the intestinal and liver injury of *Xenopus laevis* exposed to different microcystin-leucine-arginine (MC-LR) concentrations and the effects on the gut microbiota through high-throughput sequencing of 16S rDNA of the gut microbiota combined with histopathological analysis, enzyme activity determination, and qRT-PCR. Our results showed that MC-LR caused focal infiltration of inflammatory cells and increased the number of T cells and local congestion and vacuolization in *X. laevis* liver, but reduced the number, density, height, and regularity of villi. These liver and intestinal injuries became more obvious with an increase in MC-LR concentration. MC-LR significantly decreased the activities of malondialdehyde and alkaline phosphatase and the expression of *TGF-*β in the liver. Moreover, MC-LR significantly altered the gut microbiota of *X. laevis*. The relative abundance of Firmicutes and Bacteroidetes in high-concentration MC-LR groups was significantly reduced compared to that in low-concentration MC-LR groups, whereas Fusobacteria was significantly enriched. The metabolic gene composition of the gut microbiota in low-concentration MC-LR (≤5 μg/L) groups was significantly different from that in high-concentration MC-LR (≥20 μg/L) groups. These results deepen our understanding of the toxicity of MCs to aquatic organisms and assessment of the ecological risk of MCs in amphibians.

## Introduction

With the rapid development of modern industry and agricultural production, a large amount of wastewater containing nitrogen, phosphorus, and other nutrients is discharged into natural water bodies, such as rivers and lakes, resulting in increased freshwater eutrophication and frequent blue-green algal blooms (BGAB) (Ni et al., 2010; Svirčev et al., 2019). Additionally, global warming intensifies nutrient runoff and plays an important role in the occurrence and expansion of BGAB (Paerl et al., 2011; Michalak et al., 2013; Paerl and Otten, 2013). The intensity, frequency, and duration of harmful BGAB have increased worldwide (Huisman et al., 2018; Ho et al., 2019). Many blue-green algae produce cyanotoxins (Banerjee et al., 2021), which accumulate in natural water after BGAB. For instance, the average concentration of microcystins (MCs) was 11.8 μg/L (maximum concentration, 35.8 μg/L) during BGAB in Gonghu Bay, Taihu Lake, China, in 2008 (Wang et al., 2010). The peak concentration of microcystin-leucine-arginine (MC-LR) during BGAB in 2005 was 40.6 μg/L in typical artificial ponds in the Yangtze River Delta of China (Hu et al., 2018). According to the guidelines of the World Health Organization, the maximum acceptable concentration of MC-LR in drinking water is 1.0 μg/L and tolerable daily intake is 0.04 μg/kg body weight (World Health Organization, 1998). MCs can be transferred along the food chain and undergo biomagnification (Banerjee et al., 2021), thereby posing health risks to aquatic organisms, wild animals, livestock, and humans (Xiang et al., 2019). Therefore, BGAB in eutrophic water have become a major environmental and health problem worldwide (Yang et al., 2020).

MCs are the most common toxins produced by BGAB. To date, more than 240 subtypes of MCs have been reported, with MC-LR being the most common and toxic among all (Meriluoto et al., 2017). MC toxicity is organ-specific, with the liver being the most important target organ (Sun et al., 2014). Acute exposure to MCs can lead to hepatomegaly, bleeding, and even death in animals and humans, whereas long-term exposure can lead to chronic liver injury and inflammation (Massey et al., 2018). The main mechanism involves regulation of liver parameters and immunosuppression by inhibiting the production of interferon and synthesis of cytokines (Palikova et al., 2013). Inhibition of protein phosphatases (types 1 and 2A) damaged the liver, affected the redox system, and caused cellular inflammation after acute or chronic exposure (Harke et al., 2016). Moreover, MCs can damage other organs through blood circulation, and their toxic effects include apoptosis induction, cytoskeleton destruction, DNA damage, inflammation, necrosis, and oxidative stress (Liu and Sun, 2015; Zhou et al., 2015). For instance, MCs caused oxidative stress in the kidney and damaged its structure and function (Li et al., 2013). In addition, they caused oxidative stress, damage, and disruption of sex hormone levels in gonad tissues, leading to the destruction of germ cell skeleton, apoptosis, and tumor induction (Chen et al., 2013), and showed toxic effects in immune organs such as thymus and spleen, thereby affecting the immune function (Lone et al., 2016). Moreover, they could pass through the blood-brain barrier and caused functional damage to the nervous system (Kist et al., 2012). After intraperitoneal injection of MC-LR for 24 h and 90 min in Sprague-Dawley rats and Balb/c mice, respectively, significant increase in renal weight, filling of glomerular capillaries with eosinophilic fibrous substances, and moderate vacuolation of the proximal tubular epithelium along with slight dilation of tubules were observed (Hooser et al., 1989). After 13 days of acute exposure to 10 μg/kg MC-LR, the serum urea nitrogen, creatinine, and malondialdehyde (MDA) levels in male Kunming mice increased significantly (Xu, 2005). When tilapia were continuously exposed to 120 μg/kg MC-LR for 7 days, the activity of catalase (CAT), SOD, glutathione (GSH), and GR in the kidney was decreased significantly, and the dynamic redox balance was destroyed (Prieto et al., 2008). The hepatotoxicity experiments of chronic and subchronic exposure to MCs showed that chronic MC-LR immersion and exposure significantly changed the protein expression and metabolic profile of zebrafish liver through abnormal mitochondrial function, impaired aerobic respiration, interference of energy metabolism, and endoplasmic reticulum stress, eventually leading to lipid metabolism disorder (Chen L. et al., 2017). Chronic low-dose MC-LR exposure for 3 months resulted in abnormal lipid metabolism in the liver and serum of mice as well as inhibition of fatty acid β-oxidation and liver lipoprotein secretion, promoting the occurrence of liver inflammation and causing non-alcoholic steatohepatitis (He et al., 2017). However, to date, most studies on eco-toxicological risks of MCs have focused on mammals and fish (Chen et al., 2016).

Amphibians play an important role in the food web. Due to their complex life history and high skin permeability, they are more vulnerable to environmental pollution than other vertebrates (Wang et al., 2019). With amphibians being at a high level in the aquatic food chain, they have been facing a great threat of population decline and extinction in recent years. In addition to chytridiomycosis, exposure to chemical pollutants in their habitats has become the main reason for their population decline (Voyles et al., 2009; Bletz et al., 2017; Xie et al., 2019). Exposure to 2 μg/L MC-LR seriously damaged the gut tissue of *Lithobates catesbeianus* tadpoles accompanied by inflammation (He et al., 2022). MC-LR (1 μg/L) induced apoptosis in male *Rana nigromaculata* testicular cells through mitochondrial and endoplasmic reticulum pathways (Zhang et al., 2013a). Exposure of *L. catesbeianus* to 1 μg/L MC-LR for 7 days resulted in multiple organ toxicity, endocrine disorder, and impaired reproductive function (Zhang et al., 2013b; Jia et al., 2018). Prolonged exposure to BGAB (equivalent to approximately 1 μg/L MCs) caused obvious oxidative damage in the liver of *Pelophylax kl. esculentus* (Gavrilović et al., 2021). Although the gut microbiota plays an important role in amphibian digestion (Chang et al., 2016), detoxification (Zhang W. et al., 2016), development and immunity (Dawood and Koshio, 2016), and environmental adaptation (Bletz et al., 2017), the effects of MCs exposure on the expression of immune factors and composition and metabolic characteristics of the gut microbiota in amphibians have not been sufficiently studied.

Since MC-LR exposure causes an overall damage to the liver and immune system of vertebrates and triggers an inflammatory response, and gut microbiota is closely related to host metabolism and immunity, we hypothesized that MC-LR exposure might cause changes in the liver immunity and the structure and metabolic characteristics of amphibian gut microbiota, which could increase with the increase in MC-LR exposure concentration. To verify this hypothesis, we examined the intestinal and liver injury of *Xenopus laevis* under different MC-LR exposure conditions, and their effects on the structure and metabolic characteristics of the gut microbiota through high-throughput sequencing of 16S rDNA of the gut microbiota combined with histopathological analysis of the intestine and liver, and determination of liver enzyme activities and transcriptional regulation of inflammatory factors.

## Materials and Methods

### Experimental Design and Sample Collection

**FIGURE 1:**
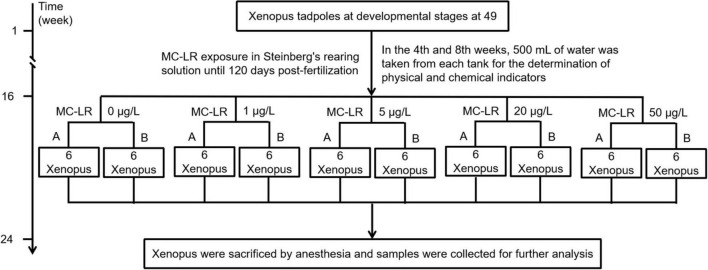
Framework shows the experimental design of the study.

The study proposal was reviewed and approved by the Animal Ethics Committee of the Qilu Normal University. Synchronously developed *X. laevis* tadpoles at the 49th stage were purchased from Nasco (Fort Atkinson, WI, United States). They were raised in Steinberg’s medium until metamorphosis into young frogs. MC-LR exposure experiment was carried out on day 120 after fertilization. Sixty synchronously developed young frogs were randomly divided into 10 aquariums (capacity, 15 L) with each aquarium having six frogs and 3 L of Steinberg’s medium containing different concentrations of MC-LR (0, 1, 5, 20, and 50 μg/L, indicated by C0, C1, C5, C20, and C50, respectively). Two parallel aquariums were set for each MC-LR concentration. One-third volume of Steinberg’s medium was replaced every 2 days with new medium containing corresponding concentration of MC-LR. Frogs were cultured at the Amphibian Breeding Laboratory of Qilu Normal University under the following conditions: temperature, 23 ± 1^°^C; humidity, 55 ± 5%; and 12 h/12 h light-dark cycle. After 8 weeks of exposure to MC-LR (Figure 1), three frogs were randomly collected from each aquarium, sacrificed, and fixed on an anatomical plate. Their abdominal cavities were immediately opened (approximately 1 cm in the middle) to collect the liver and intestine, which were divided into two parts: one part was washed with phosphate buffer and fixed in 10% formalin for tissue sectioning, and the other was stored at –80°C for RT-PCR, enzyme activity determination, and sequencing. At week 4 and 8 of the experiment, 500 mL of culture water was collected from each aquarium to monitor physicochemical factors.

MC-LR used in this study was purchased from Taiwan Algal Science Inc. Its purity (≥95%) was analyzed using high-performance liquid chromatography (HPLC; LC-10A; Shimadzu Corporation, Nakagyo-ku, Kyoto, Japan) (Moreno et al., 2004). Kits for GSH, MDA, CAT, and alkaline phosphatase (AKP) were purchased from Nanjing Jiancheng Bioengineering Institute (Nanjing, China). All other reagents were purchased from standard commercial suppliers.

### Histological, Morphological, and Immunohistochemical Analysis

Liver and gut tissues were fixed in 10% neutral buffered formalin, routinely processed, embedded in paraffin wax, sectioned (thickness, 4 μm), and stained with hematoxylin and eosin (H&E). Histopathological assessment was conducted using Pannoramic DESK + Pannoramic Scanner (3DHistech Ltd., Hungary). CD4 immunofluorescence was performed to determine the distribution of T cells in the liver and gut of *X. laevis* (Yamagami et al., 2011).

### Determination of Liver Enzyme Activities

To detect the levels of antioxidant and oxidative stress-related markers, liver tissues were homogenized in sterile normal saline in an ice bath and centrifuged at 2,500 rpm for 20 min at 4°C to collect the supernatant. Each exposure group had three biological replicates. The levels of GSH, MDA, CAT, and AKP in the supernatant were determined using commercially available kits, according to the manufacturer’s instructions (Nanjing Jiancheng, China). Coomassie brilliant blue staining was used to determine the protein content of each intestinal tissue sample (Bradford, 1976).

### qRT-PCR of Liver Immune-Associated Factors

Total RNA was isolated from liver samples using One-step RT-PCR Kit (Accurate Biology, Hunan, China), according to the manufacturer’s protocol. Total RNA concentration was determined using NanoDrop One spectrophotometer (Thermo Fisher Scientific, United States). Then, 1 μg of total RNA was reverse-transcribed into cDNA using oligo-dT primers. qRT-PCR was performed to analyze gene expression using SYBR Green Premix *Pro Taq* HS qPCR Kit (Accurate Biology, Hunan, China). Transcriptional levels of the target genes were normalized against that of glyceraldehyde-3-phosphate dehydrogenase (GAPDH). Primers (Table 1) for target genes were designed using Oligo 7.0. Primer synthesis and qRT-PCR amplification reaction conditions were consistent with those reported in our previous study (Li et al., 2022).

**TABLE 1:** Primer sequences used for qRT-PCR.

Gene name	Gene ID	Primer name	Primer sequence (5′→3′)
Glyceraldehyde-3-phosphate dehydrogenase	14433	*mGAPDH-F*	*CACAGACTTACACAGGGGTTGA*
		*mGAPDH-R*	*AGGGGTCATTGATAGCGACG*
*TNF-*α	21926	*mTnf*α*-F*	CTGTACCAGAAGCCAGAGCC
		*mTnf*α*-R*	CGATGGCGTTATCCTTGAGC
*IL-8*	20309	*mIL-8-F*	GTGTCCTGGCAATACTGGCTCTC
		*mIL-8-R*	GGGATGGATAGGCTTGCTTTCTGTC
*TGF-*β	21813	*mTGF*β*-F*	GGCTGTGGATATGGAAGAAGTCAGG
		*mTGF*β*-R*	GGCACTGTCATCTTCTCGCTGTC

### Gut Microbiota DNA Extraction and High-Throughput Sequencing

Gut microbiota DNA was extracted using PowerFecal DNA kit, as previously described (Li et al., 2022). The V4-V5 hypervariable region of 16S rDNA was amplified using the primer pair 515F/909R and sequenced using Illumina MiSeq system at Guangdong Meilikang Bio-Science Ltd., China, as previously described (Xiang et al., 2018).

Raw reads were merged using FLASH 1.2.8 software (Magoc and Salzberg, 2011) and processed using QIIME 1.9.0 pipeline (Caporaso et al., 2010), as previously described (Xiang et al., 2018; Li et al., 2022). LEfSe analysis was used to determine differences in the dominant genera (Segata et al., 2011). Functional profiles of the gut microbiota were predicted by phylogenetic investigation of communities through reconstruction of unobserved states (PICRUSt; Langille et al., 2013).

### Data Analysis

Data are presented as the mean ± standard error (SE) for each group. One-way analysis of variance (ANOVA) along with Tukey-Kramer *post-hoc* test was conducted using R 2.5.1. Non-parametric multivariate analysis of variance (PERMANOVA) was performed using the R vegan package (Dixon, 2003). Principal coordinates analysis (PCoA) was conducted using QIIME 1.9.0 pipeline. Principal component analysis (PCA) was conducted using STAMP software (Parks et al., 2014). Heatmap profiles were obtained using R pheatmap package. Differences were considered statistically significant at *p* < 0.05.

## Results

### Effect of Microcystin-LR on Histomorphology and Immunohistochemistry of the Intestine and Liver of *Xenopus laevis*

**FIGURE 2:**
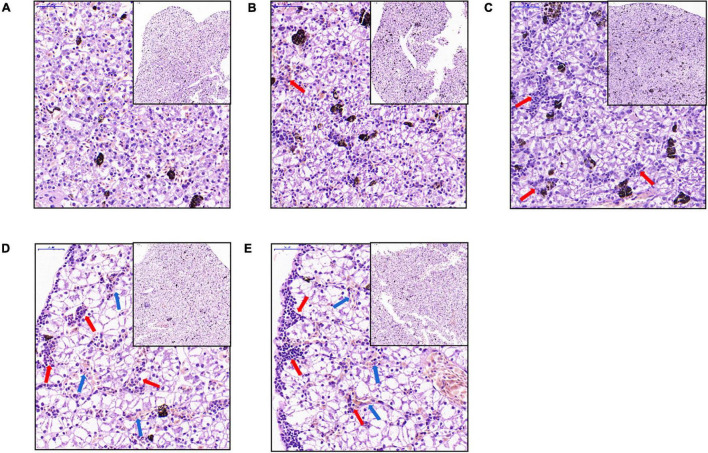
Liver microstructural changes of *Xenopus laevis* exposed to different concentrations of MC-LR. **(A–E)** Indicate the liver micrographs of *Xenopus laevis* exposed to 0, 1, 5, 20, and 50 μg/L MC-LR, respectively. Red and blue arrows indicate focal infiltration of inflammatory cells and congestion, respectively.

The liver tissues of the C0 *X. laevis* group were evenly stained. The shape and size of hepatocytes were consistent and regularly arranged. The cytoplasm was vacuolar, and no obvious inflammation was observed (Figure 2A). The liver tissues of the C1 group treated with 1 μg/L MC-LR showed multiple focal infiltrations of inflammatory cells (indicated by red arrows, Figure 2B). In the C5 (5 μg/L MC-LR), C20 (20 μg/L MC-LR), and C50 (50 μg/L MC-LR) groups, a large number of focal infiltrations of inflammatory cells was observed in the liver tissues (indicated by red arrows, Figures 2C–E). Moreover, in the C20 and C50 groups, local congestion was observed in the liver tissues (indicated by blue arrows, Figures 2D,E). These results indicate that the liver congestion and inflammation increased with an increase in MC-LR concentration.

**FIGURE 3:**
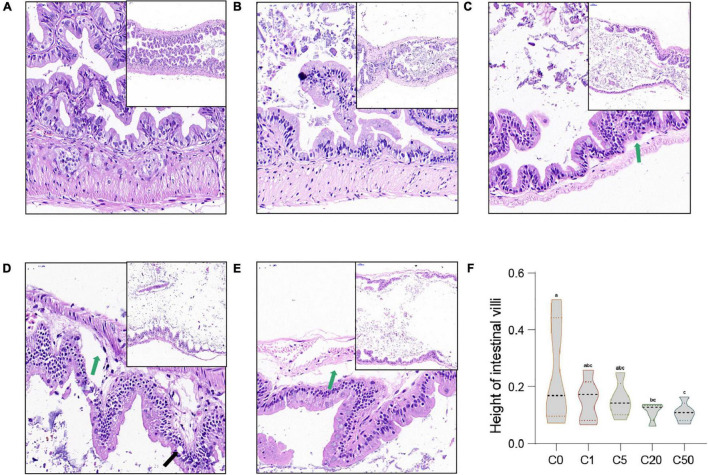
Intestinal microstructural changes of *Xenopus laevis* exposed to different concentrations of MC-LR. **(A–E)** Indicate the intestinal micrographs of *Xenopus laevis* exposed to 0, 1, 5, 20, and 50 μg/L MC-LR, respectively. **(F)** Intestinal villus height. Green and black arrows indicate the gap between the base and villi and vesicular vacuolization, respectively. Numbers after the letter C in the group names indicate MC-LR concentrations. Different letters above boxes indicate significant differences between the groups (*P* < 0.05).

In the C0 group, the intestinal villi were abundant and highly consistent. The epithelial structure of the mucosal layer was complete. The morphological structure of the epithelial cells was normal and closely arranged, with no obvious inflammation in the lamina propria (Figure 3A). In the C5, C20, and C50 groups, the number, density, and regularity of intestinal villi decreased, and cytoplasmic vacuolization (indicated by black arrow, Figure 3) and gaps between the base and villi appeared locally (indicated by green arrows, Figure 3). With an increase in MC-LR concentration, these intestinal injuries became more obvious. Moreover, the intestinal villus height in the MC-LR-exposed groups decreased with an increase in MC-LR concentration, and the values for the C20 and C50 groups were significantly different compared with the control group (C0) (*P* < 0.05; Figure 3F).

CD4 immunofluorescence of the liver T cells showed that the number of T cells in the liver increased with an increase in MC-LR concentration, and there were significant differences among the groups (*P* < 0.05; Supplementary Figure 1).

### Effect of Microcystin-LR on Liver Enzyme Activity

MDA level in the liver of *X. laevis* in the C0 group was 4.97 ± 0.24 nmol/mgprot. In the C1, C5, C20, and C50 groups, the levels were significantly lower (2.71 ± 0.12, 1.68 ± 0.11, 3.15 ± 0.19, and 3.41 ± 0.57 nmol/mgprot, respectively; *P* < 0.05; Supplementary Figure 2) than that in the C0 group.

GSH content in the C0 group was 38.49 ± 4.82 μmol/gprot; the content increased in the C5, C20, and C50 groups (59.83 ± 4.38, 44.61 ± 3.49, and 62.62 ± 1.22 μmol/gprot, respectively), but not in the C1 group (35.92 ± 2.19 μmol/gprot). Moreover, there were significant differences in GSH content in the liver among all groups (*P* < 0.05; Supplementary Figure 2B) except between the C0 and C1, C0 and C20, C1 and C20, and C5 and C50 groups.

CAT activity in the C0 liver was 44.62 ± 0.26 U/mgprot, and the activity increased with an increase in MC-LR concentration (45.23 ± 0.28, 53.73 ± 0.55, and 63.73 ± 0.35 U/mgprot in the C1, C5, and C50 groups, respectively), except the C20 group (30.80 ± 0.66 U/mgprot). Except between the C0 and C1 groups, there were significant differences in CAT level in the liver among groups (*P* < 0.05; Supplementary Figure 2C).

AKP activity in C0 liver was 304.68 ± 2.61 U/gprot, whereas it was significantly lower in the liver of C1, C5, C20, and C50 groups (53.39 ± 0.66, 116.32 ± 6.52, 138.91 ± 1.29 and 105.53 ± 2.04 U/gprot, respectively; *P* < 0.05; Supplementary Figure 2D).

### Effect of Microcystin-LR on Transcriptional Levels of Inflammatory Factors

**FIGURE 4:**
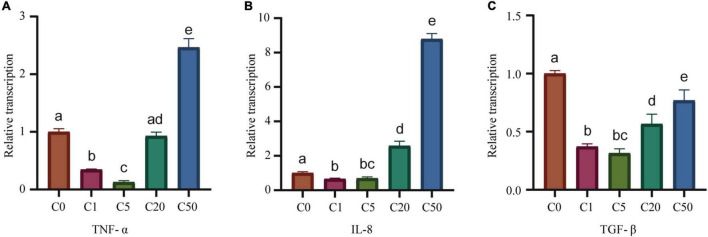
Changes in inflammatory factor gene expression in *Xenopus laevis* liver under different MC-LR concentration treatments. **(A)** TNF-α. **(B)** IL-8. **(C)** TGF-β. Numbers after the letter C in the group names indicate MC-LR concentrations. Different letters above the bars indicate significant differences between data.

Transcriptional levels of *TNF-*α and *IL-8* in the C1, C5, C20, and C50 groups were 0.35, 0.13, 0.93, and 2.46 times and 0.67, 0.70, 2.58, and 8.79 times that of C0 (Figures 4A,B), respectively. Transcriptional level of *TNF-*α and *IL-8* first decreased and then increased with an increase in MC-LR concentration. Treatment with low concentrations of MC-LR (1 and 5 μg/L) significantly reduced their transcription, whereas treatment with a high concentration of MC-LR (50 μg/L) significantly increased their transcription (*P* < 0.05; Figures 4A,B). The transcriptional levels of *TGF-*β in the liver of the C1, C5, C20, and C50 groups were 0.37, 0.32, 0.57, and 0.77 times that of C0 (Figure 4C), which was significantly lower than that of the control (C0), and there were significant differences among the groups except between the C1 and C5 groups (*P* < 0.05; Figure 4C).

### Effects of Microcystin-LR on the Gut Microbiota Structure and Metabolic Characteristics

**FIGURE 5:**
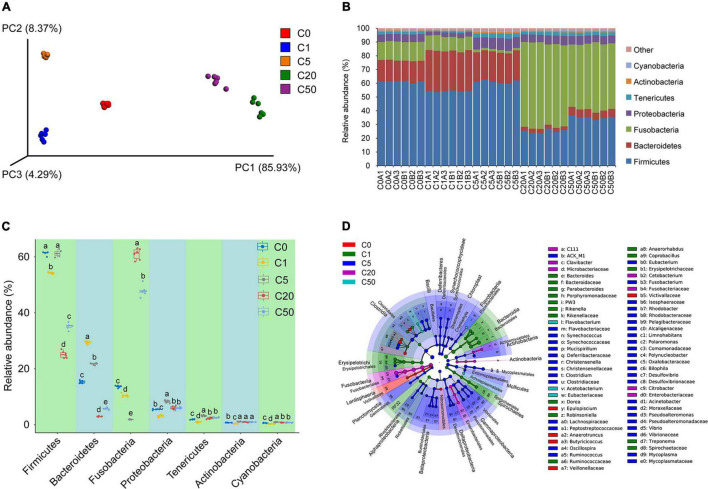
Changes in the gut microbiota composition of *Xenopus laevis* exposed to different MC-LR concentrations. **(A)** Principal coordinates analysis profile of the gut microbiota composition. **(B)** Dominant phyla of the gut microbiota. **(C)** Significant differences of the dominant phyla of the gut microbiota. **(D)** Cladogram showed the significantly different taxa of the gut microbiota. Numbers after the letter C in the group names indicate MC-LR concentrations. Different letters above boxes indicate significant differences between the groups (*P* < 0.05).

To analyze the effect of MC-LR on the gut microbiota of *X. laevis*, we sequenced 16S rRNA gene amplicons of 30 samples from the five groups (six samples in each group), and a total of 1,866,575 effective sequences were obtained. To eliminate the influence of sequencing depth on the results, 27,580 sequences were randomly resampled from each sample for subsequent analysis. A total of 25,781 operational taxonomic units (OTUs) were identified. PCoA results based on the weighted UniFrac distances showed that the samples treated with different MC-LR concentrations were clustered into five distinct groups according to MC-LR concentration (PERMANOVA, *F* = 2.640, *P* = 0.030; Figure 5A). OTU numbers of the gut microbiota in the C5, C20, and C50 groups were significantly higher than those in the C0 and C1 groups, which caused the Goods’ coverage of the gut microbiota in the C5, C20, and C50 groups to be significantly lower than those in the C0 and C1 groups (Table 2). With an increase in MC-LR concentration, the Shannon index of the gut microbiota first increased and then significantly decreased (Table 2). The Chao1 indices of the gut microbiota in the C20 and C50 groups were significantly higher than those in the C0, C1, and C5 groups, whereas the Simpson indices of the gut microbiota in the C20 and C50 groups were significantly lower than those in the C0, C1, and C5 groups (Table 2). These results implied that the presence of MC-LR in aquatic habitats significantly changed the structure of *X. laevis* gut microbiota, and with an increase in MC-LR concentration, α-diversity of the gut microbiota changed. Moreover, our results showed that the OTU number, Goods’ coverage, and Shannon index were more sensitive than Simpson and Chao1 indices in characterizing the change in α-diversity of *X. laevis* gut microbiota.

**TABLE 2:** Changes in alpha-diversity indices of *Xenopus laevis* gut microbiota exposed to different concentrations of MC-LR.

α-diversity index	C0	C1	C5	C20	C50
OTU number	1522.67 ± 61.69^b^	1395.50 ± 11.11^b^	1825.00 ± 11.23^a^	1862.50 ± 32.69^a^	1930.33 ± 48.90^a^
Goods’ coverage	0.96 ± 0.00^a^	0.96 ± 0.00^a^	0.95 ± 0.00^b^	0.95 ± 0.00^b^	0.95 ± 0.00^b^
Shannon index	5.16 ± 0.07^b^	5.26 ± 0.05^b^	5.79 ± 0.04^a^	3.90 ± 0.05^d^	4.50 ± 0.06^c^
Chao1 index	5469.84 ± 313.12^b^	5693.84 ± 171.89^b^	6014.03 ± 155.78^b^	8082.84 ± 274.07^a^	7938.73 ± 429.46^a^
Simpson index	0.92 ± 0.00^a^	0.93 ± 0.00^a^	0.93 ± 0.00^a^	0.67 ± 0.01^c^	0.78 ± 0.01^b^

*Different lowercase letters in the upper right corner indicate significant differences between data.*

**FIGURE 6:**
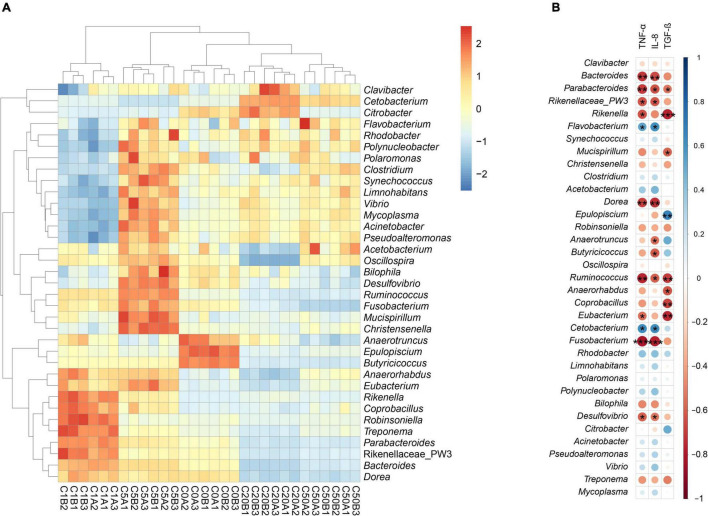
Heatmap profile shows significantly different dominant genera between *Xenopus laevis* gut microbiota with different environmental MC-LR contents **(A)** and the correlation of these genera with TNF-α, IL-8, and TGF-β **(B)**. *p < 0.05; **p < 0.01; ***p < 0.001.

Except a few sequences that could not be divided into any phyla, the other sequences were divided into 64 phyla, and Firmicutes, Bacteroidetes, Fusobacteria, Proteobacteria, Tenericutes, Actinobacteria, and Cyanobacteria dominated the gut microbiota (Figure 5B). The relative abundance of Firmicutes and Bacteroidetes in the C20 and C50 groups was significantly lower than that in the C0, C1, and C5 groups, whereas the relative abundance of Fusobacteria was significantly higher. There were also significant differences in other dominant phyla among the groups (Figure 5C). At the genus level, 1046 genera were detected, of which 66 dominated the microbiota. LEfSe results showed significant differences in all dominant genera that could be determined at the genus level. *Epulopiscium, Anaerotruncus*, and *Butyricicoccus* were significantly enhanced in the C0 group; *Bacteroides, Parabacteroides*, PW3, *Rikenella, Dorea, Robinsoniella, Anaerorhabdus, Coprobacillus*, and *Treponema* were significantly enhanced in C1; *Synechococcus, Mucispirillum, Christensenella, Clostridium, Oscillospira, Ruminococcus, Eubacterium, Fusobacterium, Rhodobacter, Limnohabitans, Polaromonas, Polynucleobacter, Bilophila, Desulfovibrio, Acinetobacter, Pseudoalteromonas, Vibrio*, and *Mycoplasma* were significantly enhanced in C5; *Clavibacter, Cetobacterium*, and *Citrobacter* were significantly enhanced in C20; and *Flavobacterium* and *Acetobacterium* were significantly enhanced in C50 (Figures 5D, 6A).

Pearson correlation analysis results showed that *Parabacteroides* and *Ruminococcus* significantly positively correlated with *TNF-*α, *IL-8*, and *TFG-*β; *Bacteroides*, Rikenellaceae PW3, *Dorea*, *Fusobacterium* and *Desulfovibrio* significantly positively correlated with *TNF-*α and *IL-8*; *Flavobacterium* and *Cetobacterium* significantly negatively correlated with *TNF-*α and *IL-8*; *Rikenella* and *Eubacterium* significantly positively correlated with *TNF-*α and *TFG-*β; *Mucispirillum*, *Anaerorhabdus* and *Coprobacillus* significantly positively correlated with *TFG-*β; and *Epulopiscium* significantly negatively correlated with *TFG-*β (Figure 6B).

**FIGURE 7:**
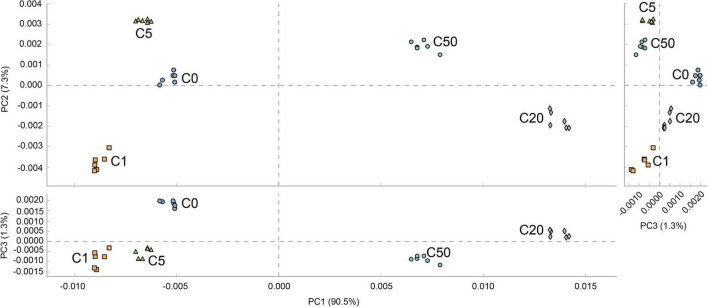
PCA profile based on metabolic characteristics of *Xenopus laevis* gut microbiota treated with different MC-LR concentrations. Numbers after the letter C in the group names indicate MC-LR concentrations.

The prediction results of the metabolic characteristics of *X. laevis* gut microbiota showed significant changes due to MC-LR exposure. Metabolic characteristics of the gut microbiota exposed to low concentrations of MC-LR (≤5 μg/L) were clearly distinguished from those exposed to high concentrations of MC-LR (≥20 μg/L) (PERMANOVA, *F* = 1121.7, *P* = 0.005; Figure 7), and analyses based on the KEGG metabolic subfamilies also exhibited similar results (Supplementary Figures 3–7). Regarding lipid metabolism, high-concentration MC-LR (≥20 μg/L) exposure significantly reduced the relative abundance of genes involved in sphingolipid metabolism and primary bile acid, secondary bile acid, and steroid hormone biosynthesis, whereas it significantly increased the relative abundance of genes involved in ether lipid, glycerolipid, arachidonic acid, and fatty acid metabolism; biosynthesis of unsaturated fatty acids; and synthesis and degradation of ketone bodies (Supplementary Figure 3). Regarding carbohydrate metabolism, high-concentration MC-LR (≥20 μg/L) exposure significantly reduced the relative abundance of genes involved in fructose, mannose, amino sugar, nucleotide sugar, galactose, starch, and sucrose metabolism and pentose and glucuronate interconversions, whereas it significantly increased the relative abundance of genes involved in glycolysis and gluconeogenesis; citrate cycle; and butanoate, propanoate, C5-branched dibasic acid, glyoxylate, dicarboxylate, inositol phosphate, and pyruvate metabolism (Supplementary Figure 4). Regarding energy metabolism, high-concentration MC-LR (≥20 μg/L) exposure significantly reduced the relative abundance of genes involved in methane metabolism but significantly increased the relative abundance of genes involved in nitrogen and sulfur metabolism (Supplementary Figure 5). Notably, high-concentration MC-LR (≥20 μg/L) exposure exhibited the opposite effect on the relative abundance of genes involved in carbon fixation in prokaryotes and photosynthetic organisms (Supplementary Figure 5). With respect to glycan biosynthesis and metabolism, high-concentration MC-LR (≥20 μg/L) exposure significantly reduced the relative abundance of genes involved in peptidoglycan and glycosphingolipid biosynthesis and glycosaminoglycan degradation, whereas it significantly increased the relative abundance of genes involved in lipopolysaccharide, N-glycan, and lipopolysaccharide biosynthesis, and glycosyltransferases (Supplementary Figure 6). In amino acid metabolism, 1 μg/L MC-LR significantly increased the relative abundance of amino acid-related enzyme genes in the gut metagenomes of *X. laevis*, whereas the relative abundance of these genes decreased significantly with an increase in MC-LR concentration (Supplementary Figure 7B). High-concentration MC-LR (≥20 μg/L) exposure significantly reduced the relative abundance of genes involved in histidine, arginine, and proline metabolism and lysine biosynthesis, whereas it significantly increased the relative abundance of genes involved in tryptophan, cysteine, methionine, phenylalanine, and tyrosine metabolism and lysine, valine, leucine, and isoleucine degradation (Supplementary Figure 7B).

## Discussion

Liver is one of the most important target organs of MCs (Sun et al., 2014). Acute toxicity of MCs destroys the structure and causes swelling, aggregation, and necrosis of hepatocytes (Yoshida et al., 1997). When cells are necrotic, integrity of the cell membrane is destroyed and the contents are released, causing an inflammatory reaction. MC-LR caused marked histopathological damage to mouse liver tissues, including lymphocyte infiltration and lipid vacuole accumulation (Fawell et al., 1999). In this study, MC-LR induced accumulation of lipid vacuoles in *X. laevis* liver, which is consistent with the results reported by Gupta et al. (2003). Moreover, accumulation became more obvious with an increase in MC-LR concentration. T lymphocytes are mainly responsible for cellular immune functions (Zapata et al., 2006). The present study showed that the number of T cells in *X. laevis* liver increased with an increase in MC-LR concentration in aquatic environment, and there were significant differences among the groups (*P* < 0.05). These results indicated that the severity of liver damage and inflammation caused by MC-LR exposure increased with an increase in MC-LR concentration.

MC-LR caused severe erosion of the intestinal villi, decrease in goblet cell size, and partial loss of microvilli (Ito et al., 2000). MC-LR also induced the release of the inflammatory cytokine TNF-α (Christen et al., 2013), altered cell membrane fluidity, and dysregulated intestinal membrane enzyme activity (Moreno et al., 2003). Moreover, the intestinal villi of zebrafish treated with MC-LR were damaged along with epithelial cell shedding and extensive cytolysis (Chen et al., 2016). In this study, our results showed that MC-LR exposure reduced the number, height, and regularity of intestinal villi as well as resulted in gap between the base and villi in *X. laevis*. With an increase in MC-LR concentration, these intestinal injuries became more obvious.

MC-dependent injuries of the liver (Massey et al., 2018), kidney (Li et al., 2013), intestine (Moreno et al., 2003), gonad (Zhao et al., 2018), and nervous system (Kist et al., 2012) are accompanied by oxidative stress. To protect the body from oxidative stress, the antioxidant defense system scavenges excessive ROS free radicals through antioxidant enzymes and non-enzymatic antioxidants such as total superoxide dismutase and GSH. GSH level was significantly increased in the intestine of *Xenopus tropicalis* treated with 0.5 μg/L MC-LR, whereas it was significantly decreased in the intestine treated with 2 μg/L MC-LR (Li et al., 2020). MDA level and enzyme activity and transcriptional levels of the antioxidants CAT were increased in zebrafish ovaries injected with MC-LR (Qiao et al., 2013), indicating the occurrence of oxidative stress. Our results showed that GSH content was significantly increased in the liver of *X. laevis* treated with 5, 20, and 50 μg/L MC-LR, but not in those treated with 1 μg/L MC-LR. These results indicate that low concentration of MC-LR improved detoxification ability of the antioxidant defense system in *X. laevis* intestine. However, exposure to a high concentration of MC-LR decreased the ability of the antioxidant defense system in *X. laevis* intestine. MC-LR exposure caused an initial increase and then decrease in the activity of AKP in the hepatopancreas of *Penaeus vannamei* (Chen Y. Y. et al., 2017). In this study, similar changes were observed for CAT content in *X. laevis* liver. Except the group exposed to 20 μg/L MC-LR, CAT content increased with an increase in MC-LR concentration in the other groups. However, in contrast, MDA and AKP contents in *X. laevis* liver exposed to MC-LR were significantly decreased compared with the control (*P* < 0.05; Supplementary Figure 2). This was probably because the responses of different species to MC-LR vary based on their toxicity sensitivities, and the mechanism remains to be further investigated.

TNF-α, IL-1β, and IL-8 are important markers of inflammatory responses, and their upregulation is usually associated with inflammatory diseases (Papadakis and Targan, 2000), whereas IL-10, TGF-β, and other anti-inflammatory cytokines inhibit the production of pro-inflammatory cytokines and prevent inflammation (Chen and Manning, 1996). In this study, the expression of *TNF-*α and *IL-8* in *X. laevis* liver first decreased and then increased with an increase in MC-LR exposure concentration, and their expression in *X. laevis* liver exposed to low-concentration MC-LR (1 and 5 μg/L) was significantly reduced, whereas their expression in *X. laevis* liver exposed to high-concentration MC-LR (50 μg/L) was significantly increased comparing to healthy control (*P* < 0.05; Figures 4A,B). After MC-LR exposure, the expression of *TGF-*β, an anti-inflammatory factor, was significantly lower than that of the control group (*P* < 0.05; Figure 4C), which was consistent with previous reports (Li et al., 2019; Ding et al., 2021). These results implied that MC-LR stimulated the release of pro-inflammatory cytokines and induced an inflammatory response, and high concentrations of MC-LR are immunotoxic to *X. laevis*.

The effect of MC-LR on the gut microbiota diversity remains controversial. Zhang et al. (2020) reported that MC-LR altered the gut microbial composition of freshwater crayfish (*Procambarus clarkii*), reducing its richness and diversity. In contrast, Chen et al. (2015) reported that MC-LR increased microbiota richness in mouse cecum and colon. Our results showed that OTU numbers of the gut microbiota in the C5, C20, and C50 groups were significantly higher than those in the C0 and C1 groups, which caused the Good’s coverage of the gut microbiota in the C5, C20, and C50 groups to be significantly lower than those in the C0 and C1 groups. The Chao1 indices of the gut microbiota in the C20 and C50 groups were significantly higher than those in the C0, C1, and C5 groups. With an increase in MC-LR concentration, the Shannon index of the gut microbiota first increased and then significantly decreased, whereas the Simpson indices of the gut microbiota in the C20 and C50 groups were significantly lower than those in the C0, C1, and C5 groups. It was speculated that the variation in results might be attributed to differences in species, administration methods, or MC-LR doses.

As a key factor in the regulation of the immune system, the gut microbiota is a rich source of pro-inflammatory factors (Cani et al., 2007; Li et al., 2020). MC-LR probably induced inflammation of the peripheral tissues by changing the composition of the gut microbiota, resulting in lipid metabolism disorder (Zhang Z. Y. et al., 2016). Local expansion of Fusobacteria activates the host inflammatory response and affected the barrier functions (Kostic et al., 2013), and Proteobacteria are mainly responsible for the utilization of amino acids in the intestine and regulation of intestinal inflammation (Shin et al., 2015). Actinobacteria, one of the four most abundant phyla in the gut microbiota, play an important role in the steady-state regulation of the intestinal barrier (Binda et al., 2018). In our study, Fusobacteria, Proteobacteria, and Actinobacteria were detected as dominant phylums in the gut of *X. laevis* (Figure 5B). The relative abundance of Fusobacteria in the gut microbiota of the C20 and C50 groups was significantly higher than that in the C0, C1, and C5 groups. The previous study has shown that subacute MC-LR treatment impairs the diversity of Bacteroidetes (Chen et al., 2015), and metagenomic analyses of mouse gut microbiota revealed that MC-LR exposure increased abundant ratio of Firmicutes vs. Bacteroidetes in the gut (Zhang Z. Y. et al., 2016). Interestingly, we found that the relative abundance of Firmicutes and Bacteroidetes in the C20 and C50 groups were significantly lower than those in the C0, C1, and C5 groups in *X. laevis* with subchronic MC-LR exposure. We speculate that it could be attribute to differences in route and magnitude of exposure to the toxin. Additionally, significant differences were observed in Tenericutes and Cyanobacteria abundance among the groups (Figure 5C). The above results suggest that the changes in *X. laevis* gut microbiota caused by different concentrations of MC-LR are potentially correlated with host inflammation.

As one of the most important symbiotic conditional fish pathogens widely distributed in freshwater, *Flavobacterium columnare* affects the health status of wild and cultured fish and causes profound loss in aquaculture (Arias et al., 2004; Suomalainen et al., 2009). Highly toxic strains of *F. columnare* caused death of silver salmon fry (*Oncorhynchus kisutch*) within 24 h (Rucker et al., 1953). The expression levels of IL-1β and IL-6 in mouse brain were positively correlated with the abundance of *Flavobacterium* (Szyszkowicz et al., 2017). This indicates that the abundance of *Flavobacterium* reflects the inflammatory status of the host. Simultaneously, as a gram-negative bacterium, an increase in its abundance aggravates the level of lipopolysaccharide (LPS) and inflammatory responses (Cani et al., 2007). In this study, *Flavobacterium* was identified as a dominant genus and was significantly enriched in the gut microbiota of *X. laevis* exposed to 50 μg/L MC-LR. These results indicated that 50 μg/L MC-LR exposure severely damaged the structure and increased inflammation of the gut microbiota of *X. laevis*.

*Vibrio* is the main bacterial pathogen in patients with acute diarrhea (Wang et al., 2021). *Bilophila* and *Ruminococcus gnavus* are pathogens that cause inflammatory bowel disease (IBD) (Hall et al., 2017). *R. gnavus* contributed to significant upregulation of oxidative stress-related pathways in the gut microbiota of patients with IBD (Kumar et al., 2016; Hall et al., 2017). *Pseudoalteromonas* was positively correlated with the occurrence of asthma, rhinitis, and rhinoconjunctivitis. This is probably because *Pseudoalteromonas* produces cyclodigiosin hydrochloride, an immunosuppressant that inhibits the proliferation of T cells. Immunosuppression overactivates the type 2 response, which in turn increases the risk of allergies and asthma (Spellberg and Edwards, 2001). *Acinetobacter* is a common opportunistic pathogen that causes serious infections (Munoz-Price and Weinstein, 2008). Increased *Clostridia* levels are associated with inflammation (Pearson-Leary et al., 2019). An increase in the abundance of *Desulfovibrio* is closely related to metabolic diseases (Petersen et al., 2019). *Fusobacterium* induced inflammation through TNF-α and NF-κB in an *in vitro* cultured colorectal cancer cell line (Salvucci et al., 2021). In steatosis, steatohepatitis, and hepatocellular carcinoma, the abundance of *Mucispirillum* increases with an increase in the degree of inflammation (Zhang et al., 2021). In this study, *Mucispirillum*, *Clostridium*, *Ruminococcus*, *Fusobacterium*, *Bilophila, Desulfovibrio*, *Acinetobacter*, *Pseudoalteromonas*, and *Vibrio* were significantly enriched in the C5 group, indicating that 5 μg/L MC-LR exposure caused a large number of pathogenic bacteria to colonize the gut microbiota of *X. laevis* and produce a series of pro-inflammatory reactions. *Bacteroides* reduced the L-glutathione-to-glutathione ratio and hepatocyte apoptosis, and alleviated liver injury by inhibiting the expression of CD95 and CD95/CD95L signaling in mouse hepatocytes (Wang et al., 2022). Short-chain fatty acids produced by *Rikenella* improved the intestinal barrier by promoting cell differentiation and tight junctions (Cani et al., 2009). *Parabacteroides* and *Coprobacillus* were positively correlated with the severity of COVID-19 disease (Zuo et al., 2020; Schult et al., 2022). *Dorea* and *Treponema* were significantly enriched in the gut microbiota of patients with autism and endometrial cancer, respectively (Walther-António et al., 2016; Strati et al., 2017). Our results showed that *Bacteroides* and *Rikenella* were significantly enriched in the C1 group, implying that they play an important role in maintaining healthy energy metabolism and immune function in *X. laevis*. However, the enrichment of *Parabacteroides*, *Coprobacillus*, *Dorea*, and *Treponema* suggested that although water containing 1 μg/L MC-LR complied with the guidelines of the World Health Organization (1998), MC-LR presence still had a certain impact on the gut microbiota of *X. laevis*. This is probably due to the complex life history and high skin permeability of amphibians (Wang et al., 2019). These results indicated that subchronic exposure to MC-LR significantly affected the gut microbiota structure of *X. laevis*.

Previous studies have mainly performed acute toxicity experiments. However, because aquatic organisms are exposed to natural water for a long time, the acute poisoning threshold concentration of MCs for them is often lower that the concentration in the environment. Therefore, chronic or subchronic toxicity experiments are valuable for ecological safety assessment.

## Conclusion

MC-LR caused focal infiltration of inflammatory cells and increased the number of T cells and local congestion and vacuolization in the liver of *X. laevis*. Simultaneously, MC-LR reduced the number, density, height, and regularity of intestinal villi and led to gaps between the intestinal villi and basal layer. Moreover, with an increase in MC-LR concentration, these injuries to the liver and intestine became more obvious. MC-LR significantly decreased the levels of MDA and AKP and the expression of *TGF-*β in the liver, whereas the expression of *TNF-*α and *IL-8* first decreased and then increased with an increase in MC-LR concentration. MC-LR significantly altered the structure and metabolic characteristics of the gut microbiota of *X. laevis*. The OTU numbers of the gut microbiota in the C5, C20, and C50 groups were significantly higher than those in the C0 and C1 groups. The relative abundance of Firmicutes and Bacteroidetes in the C20 and C50 groups were significantly lower than those in the C0, C1, and C5 groups, whereas the relative abundance of Fusobacteria was significantly higher. The metabolic gene composition of the gut microbiota of *X. laevis* exposed to low MC-LR concentration (≤5 μg/L) was significantly different from that of *X. laevis* exposed to high MC-LR concentration (≥20 μg/L).

## Data Availability Statement

The datasets presented in this study can be found in online repositories. The names of the repository/repositories and accession number(s) can be found in the article/Supplementary Material.

## Ethics Statement

The animal study was reviewed and approved by the Biological and Medical Ethics Committee of Qilu Normal University.

## Author Contributions

JL designed the experiments. JL, HS, and SL performed the animal experiments. JL and CW conducted gut microbiota analysis. HS, SL, and YC measured biochemical data and conducted immunohistochemical analysis. JL, HS, and CW wrote the draft. All authors revised and approved the final version of the manuscript.

## Conflict of Interest

The authors declare that the research was conducted in the absence of any commercial or financial relationships that could be construed as a potential conflict of interest.

## Publisher’s Note

All claims expressed in this article are solely those of the authors and do not necessarily represent those of their affiliated organizations, or those of the publisher, the editors and the reviewers. Any product that may be evaluated in this article, or claim that may be made by its manufacturer, is not guaranteed or endorsed by the publisher.

## Supplementary Material

Supplementary Figure 1 Number of T cells in the liver of *Xenopus laevis* exposed to different MC-LR concentrations. **(A–E)** Indicate the immunofluorescence liver micrographs of *Xenopus laevis* exposed to 0, 1, 5, 20, and 50 μg/L MC-LR, respectively. **(F)** Number of T cells in *Xenopus laevis* liver. Different letters above boxes indicate significant differences between the groups (*P* < 0.05).

Supplementary Figure 2 Changes in the content of MAD **(A)**, GSH **(B)**, CAT **(C)**, and AKP **(D)** in the liver of *Xenopus laevis* under different MC-LR treatments. Numbers after the letter C in the group names indicate MC-LR concentrations. Different letters above the bars indicate significant differences between data.

Supplementary Figure 3 PCA **(A)** and heatmap **(B)** profiles showed changes of genes participating in the lipid metabolism. ^**^*p* < 0.01; ^***^*p* < 0.001.

Supplementary Figure 4 PCA **(A)** and heatmap **(B)** profiles showed changes of genes participating in the carbohydrate metabolism. ^***^*p* < 0.001.

Supplementary Figure 5 PCA **(A)** and heatmap **(B)** profiles showed changes of genes participating in the energy metabolism. ^**^*p* < 0.01; ^***^*p* < 0.001.

Supplementary Figure 6 PCA **(A)** and heatmap **(B)** profiles showed changes of genes participating in the glycan metabolism. **p* < 0.05; ^***^*p* < 0.001.

Supplementary Figure 7 PCA **(A)** and heatmap **(B)** profiles showed changes of genes participating in the amino acid metabolism. ^***^*p* < 0.001.

Supplementary Table 1 Source data for the intestinal villus height and percentage of T cells in the liver of Xenopus laevis.

Supplementary Table 2 Source data for the merged sequences, and original microscopy images in the Figures 2–4.

Supplementary Table 3 Source data for the inflammatory factor gene expression in Xenopus laevis liver.

Supplementary Table 4 Source data for the content of MAD, GSH, CAT, and AKP in Xenopus laevis liver.

Supplementary Table 5 Source data for the dominant phyla and genus of the Xenopus laevis gut microbiota.

Supplementary Table 6 Source data for the metabolic characteristics of the Xenopus laevis gut microbiota.
