# Supplementary figures and images for: Subchronic Toxicity of Microcystin-LR on Young Frogs (Xenopus laevis) and Their Gut Microbiota
Source: Front Microbiol. 2022 May 12;13:895383. doi: 10.3389/fmicb.2022.895383 (PMC9134123; doi:10.3389/fmicb.2022.895383)

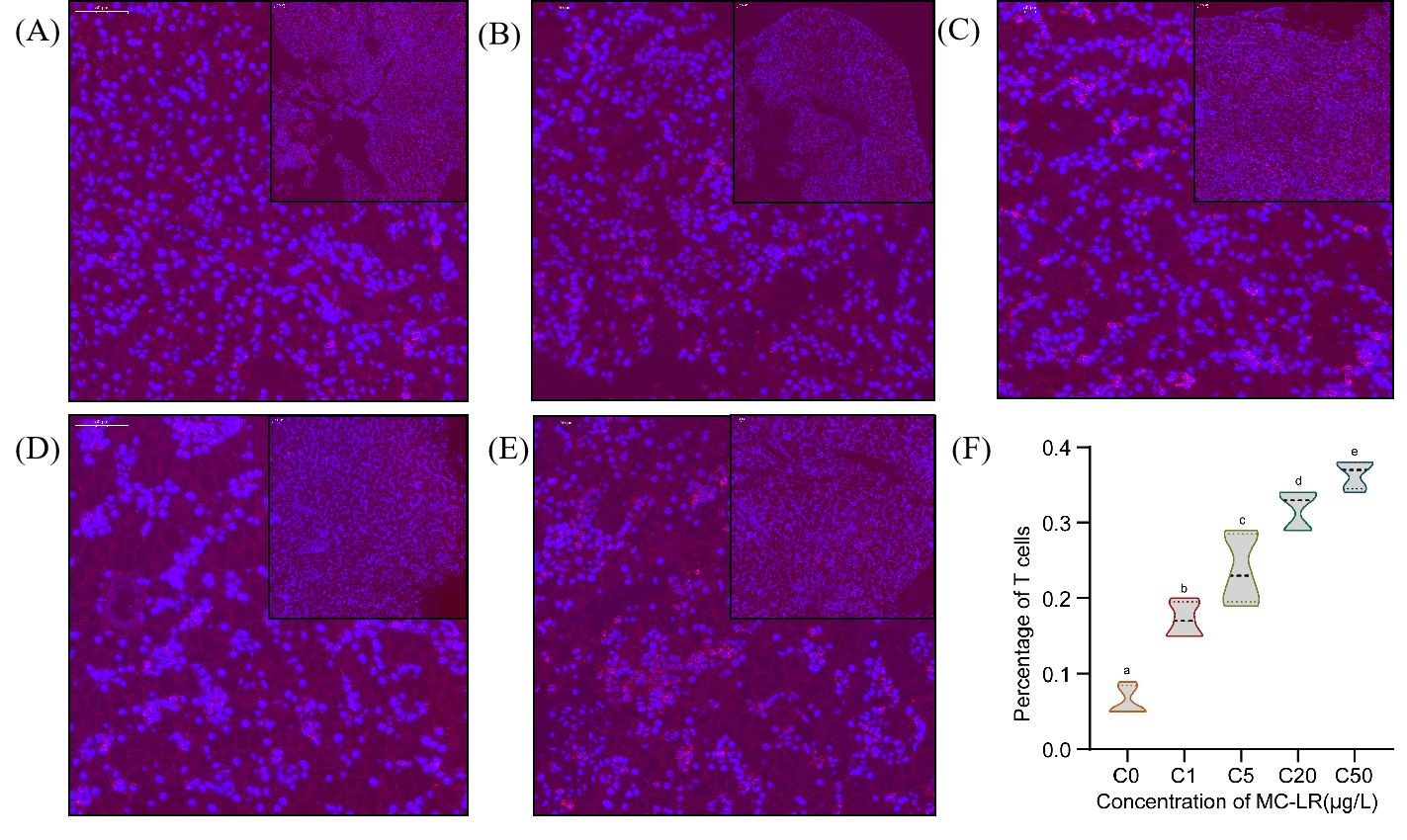

Supplement: Supplementary Figure 1 — Number of T cells in the liver of Xenopus laevis exposed to different MC-LR concentrations. (A–E) Indicate the immunofluorescence liver micrographs of Xenopus laevis exposed to 0, 1, 5, 20, and 50 μg/L MC-LR, respectively. (F) Number of T cells in Xenopus laevis liver. Different letters above boxes indicate significant differences between the groups (P < 0.05). [file Image_1.TIF]

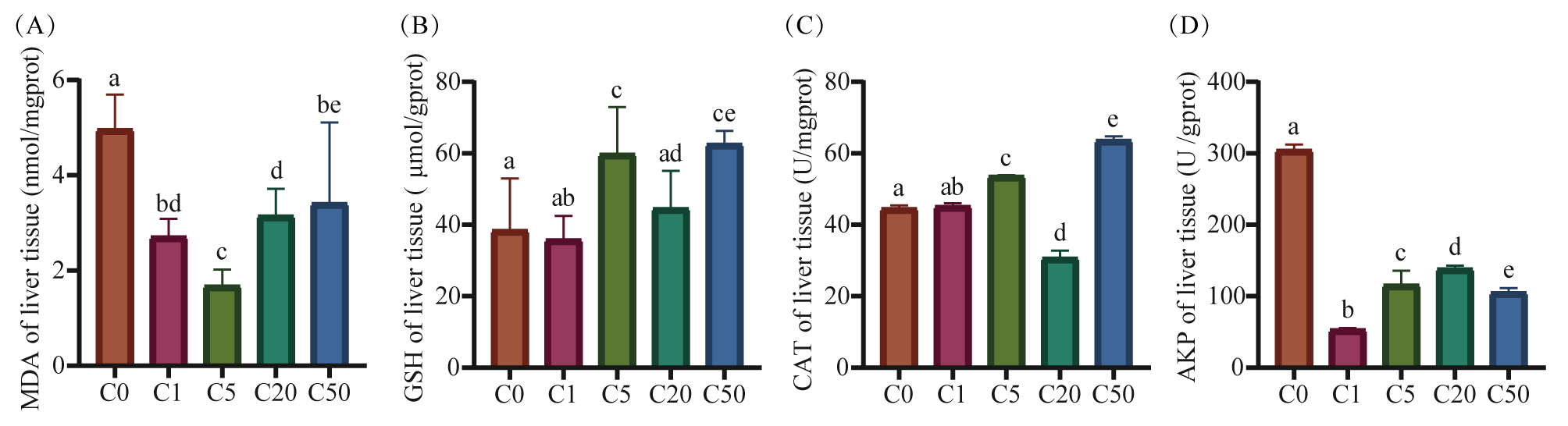

Supplement: Supplementary Figure 2 — Changes in the content of MAD (A), GSH (B), CAT (C), and AKP (D) in the liver of Xenopus laevis under different MC-LR treatments. Numbers after the letter C in the group names indicate MC-LR concentrations. Different letters above the bars indicate significant differences between data. [file Image_2.TIF]

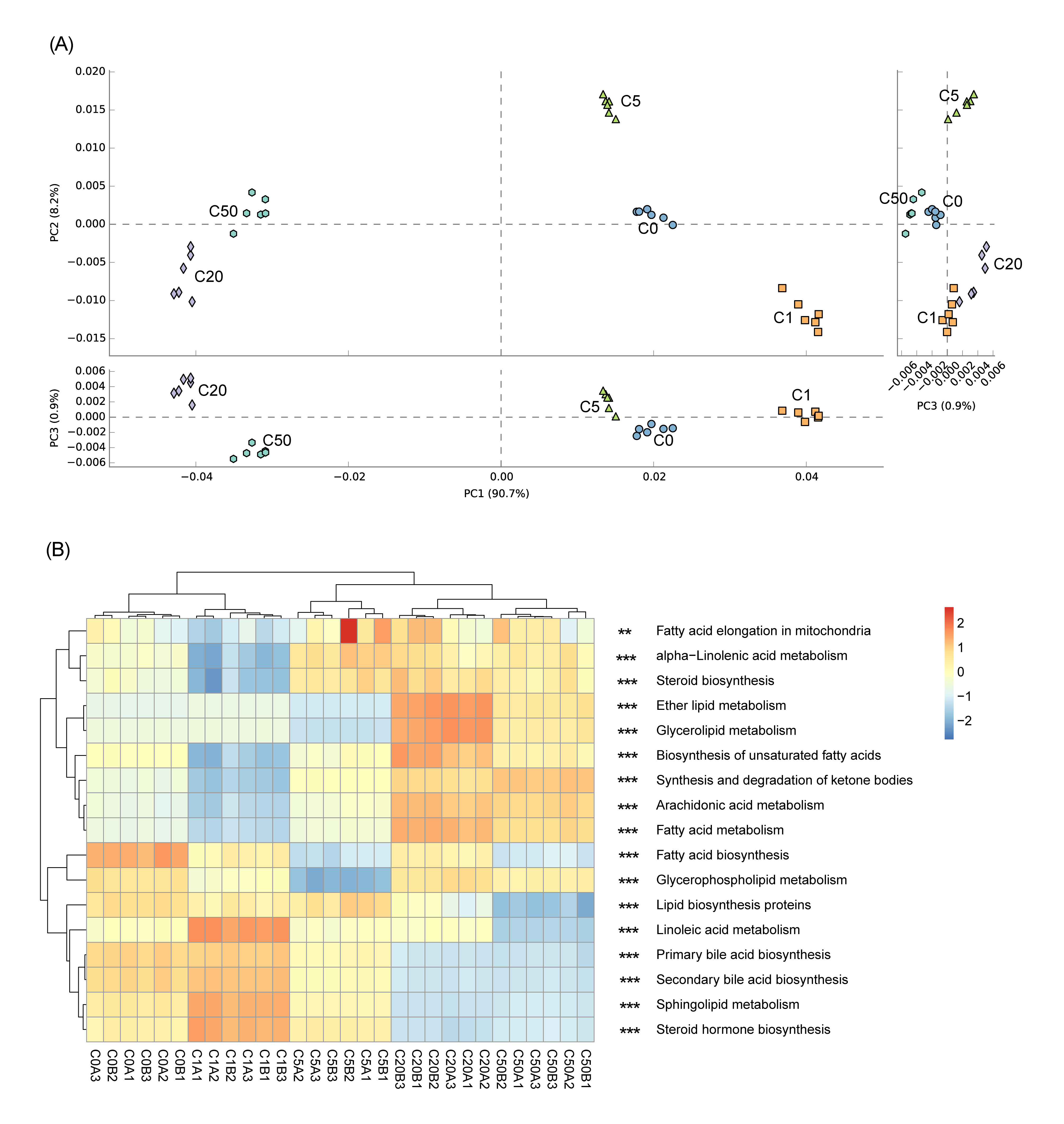

Supplement: Supplementary Figure 3 — PCA (A) and heatmap (B) profiles showed changes of genes participating in the lipid metabolism. **p < 0.01; ***p < 0.001. [file Image_3.TIF]

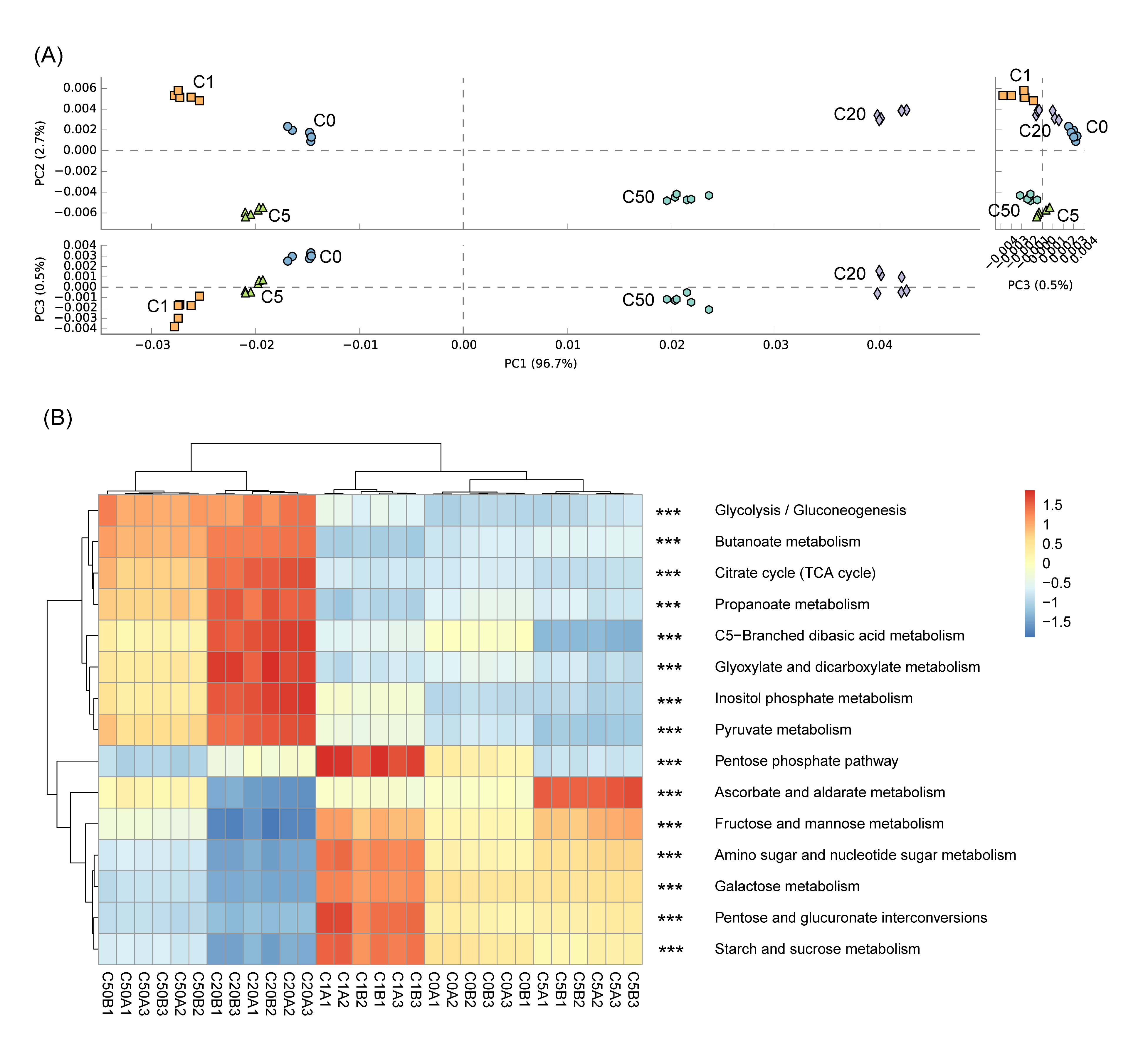

Supplement: Supplementary Figure 4 — PCA (A) and heatmap (B) profiles showed changes of genes participating in the carbohydrate metabolism. ***p < 0.001. [file Image_4.TIF]

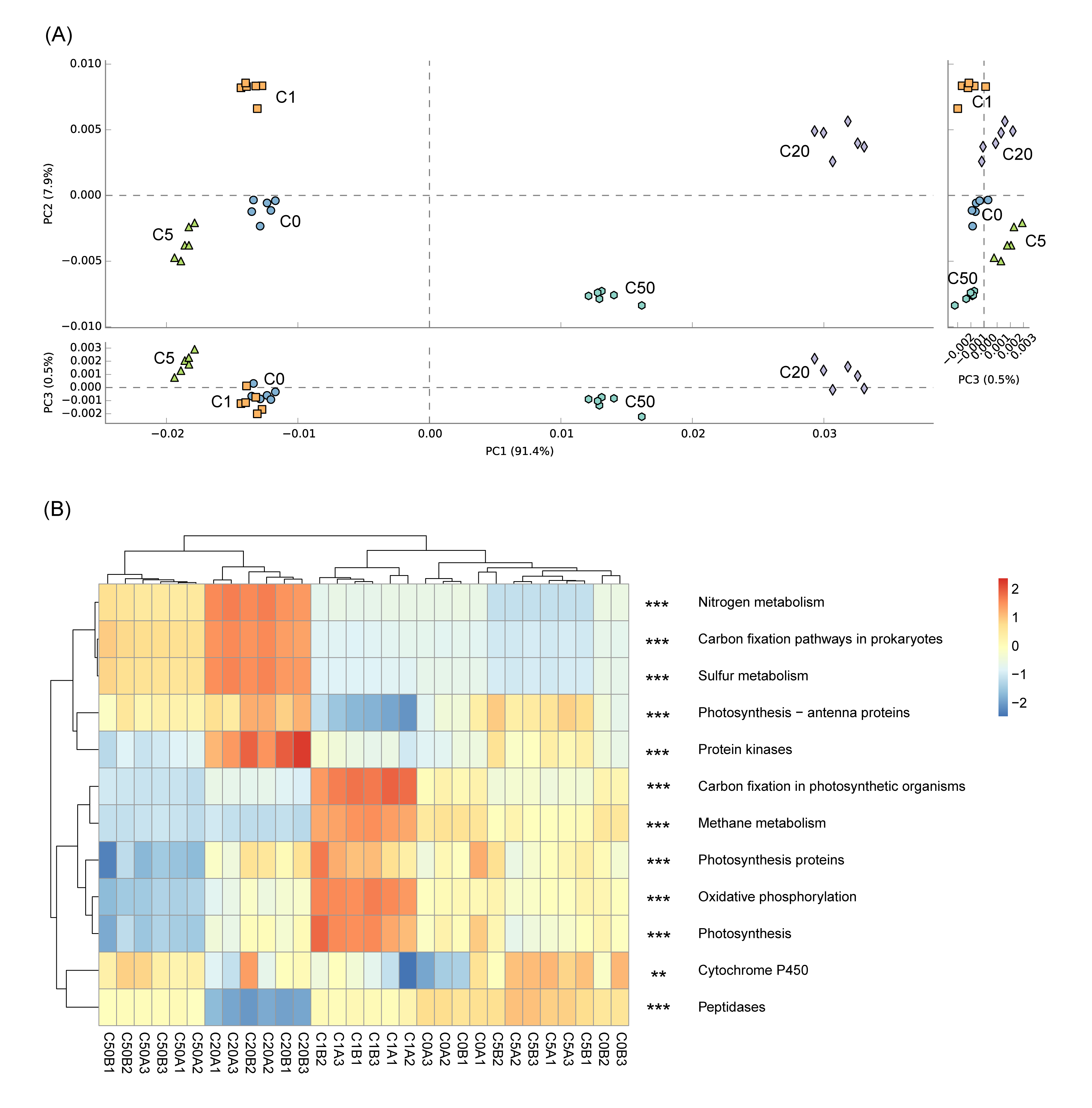

Supplement: Supplementary Figure 5 — PCA (A) and heatmap (B) profiles showed changes of genes participating in the energy metabolism. **p < 0.01; ***p < 0.001. [file Image_5.TIF]

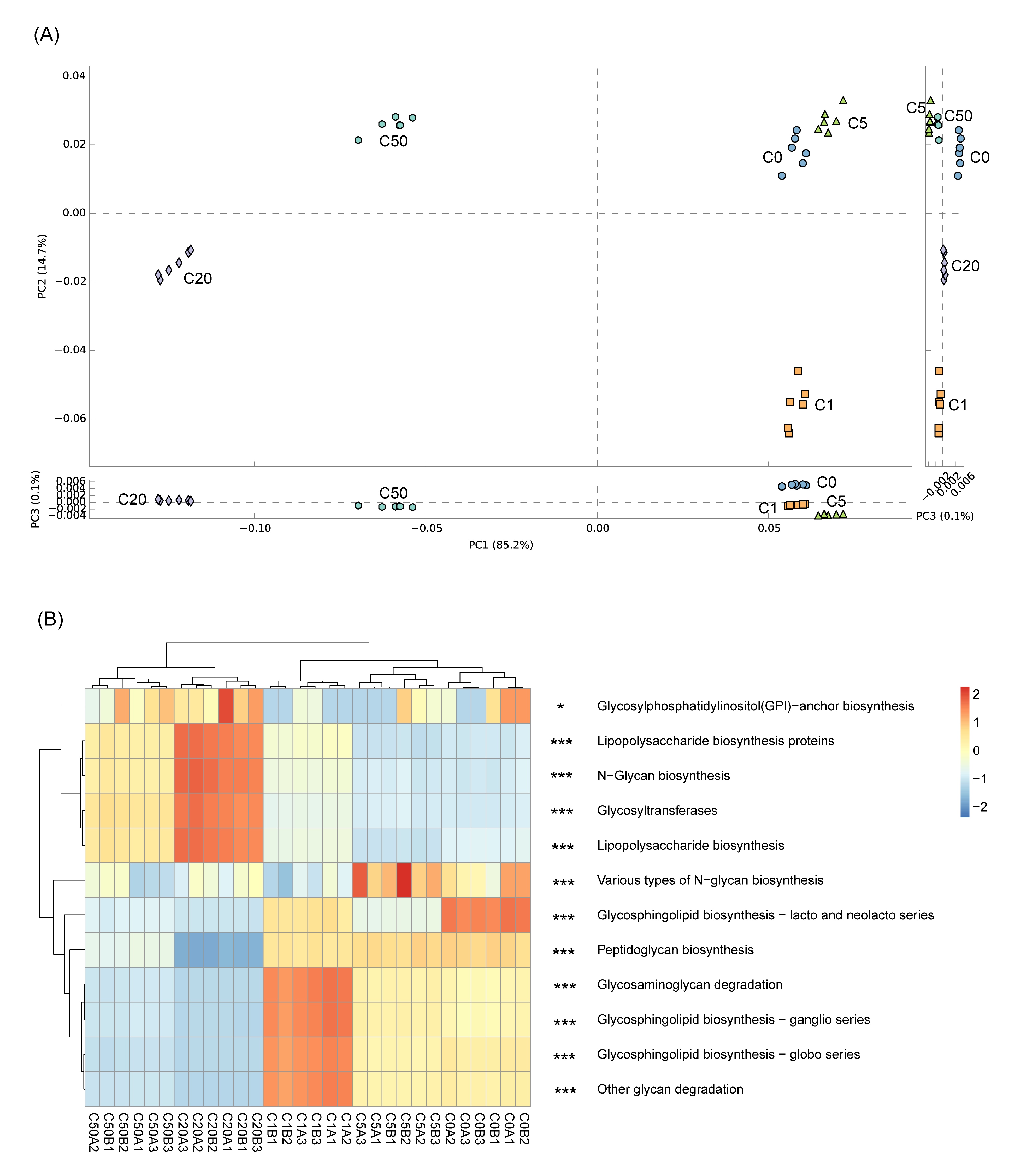

Supplement: Supplementary Figure 6 — PCA (A) and heatmap (B) profiles showed changes of genes participating in the glycan metabolism. *p < 0.05; ***p < 0.001. [file Image_6.TIF]

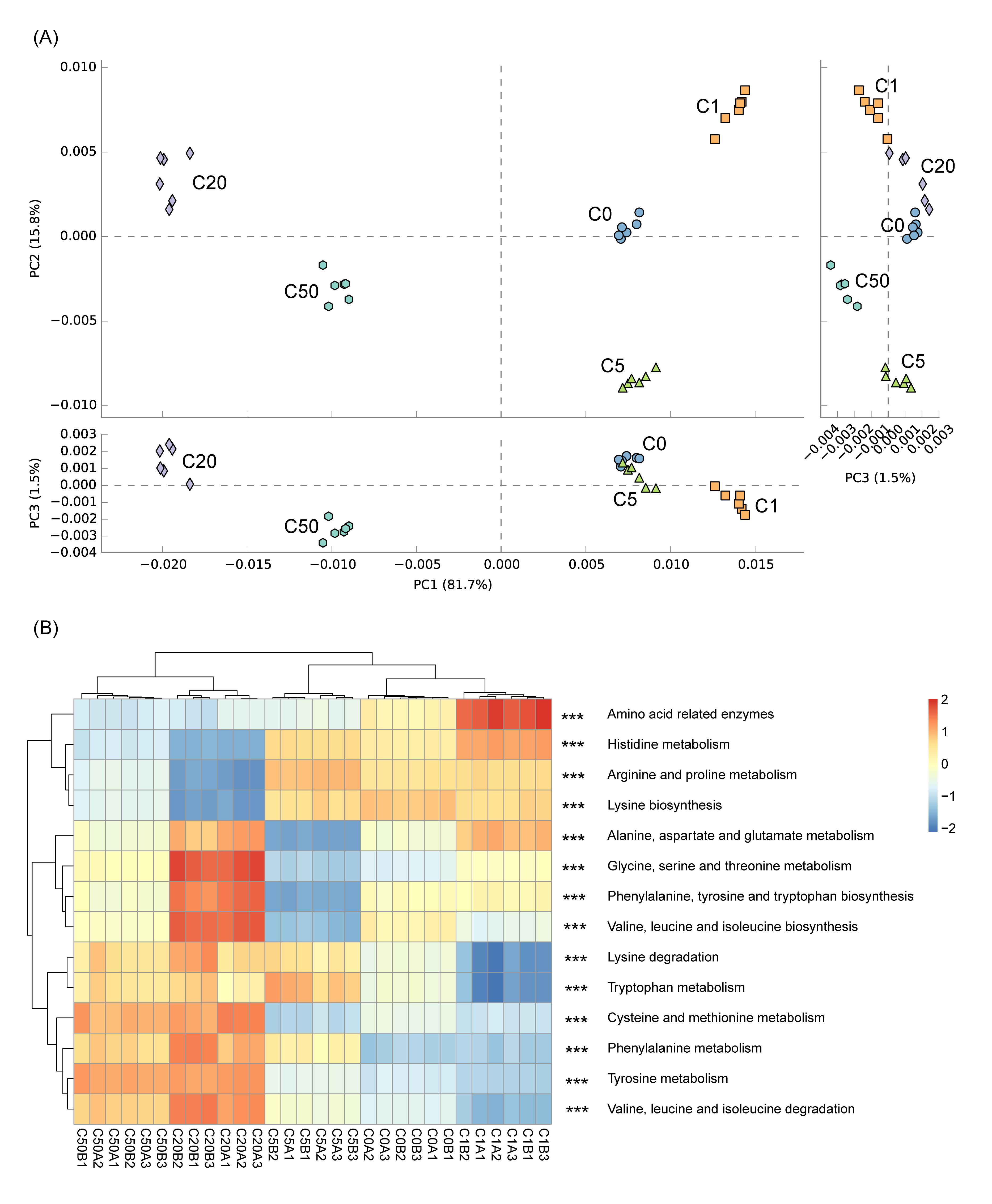

Supplement: Supplementary Figure 7 — PCA (A) and heatmap (B) profiles showed changes of genes participating in the amino acid metabolism. ***p < 0.001. [file Image_7.TIF]
