# Supplementary material for: Subchronic Toxicity of Microcystin-LR on Young Frogs (Xenopus laevis) and Their Gut Microbiota
Source: Front Microbiol. 2022 May 12;13:895383. doi: 10.3389/fmicb.2022.895383 (PMC9134123; doi:10.3389/fmicb.2022.895383)
Supplement: Supplementary Table 2 — Source data for the merged sequences, and original microscopy images in the Figures 2–4. [file Table_2.DOCX]

The merged sequences were deposited in the Genome Sequence Archive with the access number CRA009234 (https://bigd.big.ac.cn/gsa/browse/CRA006264).

Download links of original microscopy images in the Figure 2, Figure 3, and Figure 4

Figure 2

C0_10.0X

https://www.jianguoyun.com/p/DRVZkkQQm5iLChi_jLIE

C0_40.0X

https://www.jianguoyun.com/p/DRVZkkQQm5iLChi_jLIE

C1_10.0X

https://www.jianguoyun.com/p/DfKkvFcQm5iLChjGjLIE

C1_40.0X

https://www.jianguoyun.com/p/Db_--OkQm5iLChjHjLIE

C5_10.0X

https://www.jianguoyun.com/p/DUonXo4Qm5iLChjIjLIE

C5_40.0X

https://www.jianguoyun.com/p/DRxHC9QQm5iLChjLjLIE

C20_10.0X

https://www.jianguoyun.com/p/DZ7nNwUQm5iLChjNjLIE

C20_40.0X

https://www.jianguoyun.com/p/DSP4R68Qm5iLChjPjLIE

C50_10.0X

https://www.jianguoyun.com/p/DVazTjoQm5iLChjcjLIE

C50_40.0X

https://www.jianguoyun.com/p/DWkazcgQm5iLChjdjLIE

Figure 3

C0_10.0X

https://www.jianguoyun.com/p/DU7rHJMQm5iLChjejLIE

C0_40.0X

https://www.jianguoyun.com/p/DVnyFz4Qm5iLChjfjLIE

C1_10.0X

https://www.jianguoyun.com/p/DdJIxssQm5iLChjmjLIE

C1_40.0X

https://www.jianguoyun.com/p/DUn7ZScQm5iLChjnjLIE

C5_10.0X

https://www.jianguoyun.com/p/DbmrkYkQm5iLChjojLIE

C5_40.0X

https://www.jianguoyun.com/p/Dful48AQm5iLChjpjLIE

C20_10.0X

https://www.jianguoyun.com/p/DchsrZEQm5iLChjrjLIE

C20_40.0X

https://www.jianguoyun.com/p/DeY12xkQm5iLChjsjLIE

C50_10.0X

https://www.jianguoyun.com/p/DaBoyv8Qm5iLChjtjLIE

C50_40.0X

https://www.jianguoyun.com/p/DdZTIGgQm5iLChjujLIE

Figure 4

C0_10.0X

https://www.jianguoyun.com/p/DVQnvPkQm5iLChjwjLIE

C0_40.0X

https://www.jianguoyun.com/p/DYGchVMQm5iLChjxjLIE

C1_10.0X

https://www.jianguoyun.com/p/DYGchVMQm5iLChjxjLIE

C1_40.0X

https://www.jianguoyun.com/p/DaOg8K4Qm5iLChjzjLIE

C5_10.0X

https://www.jianguoyun.com/p/DeWGLXEQm5iLChj0jLIE

C5_40.0X

https://www.jianguoyun.com/p/DRXz9L4Qm5iLChj1jLIE

C20_10.0X

https://www.jianguoyun.com/p/DV6hqHUQm5iLChj2jLIE

C20_40.0X

https://www.jianguoyun.com/p/DVm5oQEQm5iLChj3jLIE

C50_10.0X

https://www.jianguoyun.com/p/DaqENWEQm5iLChj4jLIE

C50_40.0X

https://www.jianguoyun.com/p/DbHC8tUQm5iLChj5jLIE
